# Research note: Reference genes selection for gene expression analyses in reproductive turkey (*Meleagris gallopavo*) with yellow semen syndrome

**DOI:** 10.1016/j.psj.2025.105093

**Published:** 2025-03-24

**Authors:** Ewa M. Drzewiecka, Ewa Liszewska, Krzysztof Kozłowski, Andrzej Ciereszko, Mariola Słowińska

**Affiliations:** a Team of Gamete Biology, Institute of Animal Reproduction and Food Research, Polish Academy of Sciences in Olsztyn, Trylińskiego 18, 10-683 Olsztyn, Poland; b Department of Poultry Science and Apiculture, Faculty of Animal Bioengineering, University of Warmia and Mazury in Olsztyn, Oczapowskiego 5, 10-719 Olsztyn, Poland

**Keywords:** Turkey, Yellow semen syndrome, qPCR, Reference gene

## Abstract

Molecular biology techniques, including qPCR, are reliable, powerful, and commonly used tools for diagnostic purposes. qPCR might be useful for determining the etiology of yellow semen syndrome (**YSS**), an endemic condition in the turkey population, leading to decreased reproductive potential of this species. The current study aimed to evaluate the most accurate qPCR internal controls that might be used as first-choice-reference genes in studies on the reproductive tract, liver, and immune tissues and cells of adult male turkey breeders. RNA was isolated from testis, epididymis, ductus deferens, liver, thymus, bursa of Fabricius, spleen, whole blood, and peripheral blood mononuclear cells (**PBMC**) from healthy adult individuals producing white semen (**WS**, *n* = 6) and with yellow semen syndrome (YSS, *n* = 6). The expression of seven commonly used housekeeping genes, i.e., *glyceraldehyde-3-phosphate dehydrogenase* (***GAPDH***), *actin beta* (***ACTB***), *phosphoglycerate kinase* (***PGK1***), *60S ribosomal protein L13* (***RPL13***)**,***ribosomal protein L19* (***RPL19***), *transferrin receptor protein* (***TFRC***), and *vimentin* (***VIM***) was tested with qPCR followed by computational calculation of these genes' expression stability in selected tissues/cells. For YSS studies we recommend using *ACTB/GAPDH* gene pair as a reference for male reproductive organs i.e. testis, epididymis, and ductus deferens as well as in the liver, *RPL13/RPL19* for central immune organs, i.e. bursa of Fabricius and thymus, and *RPL13/VIM, ACTB/RPL19*, and *PGK1/RPL19* in the spleen, whole blood, and PBMC research, respectively. A careful investigation of the stability of these genes in each following experiment is required to maintain high-accuracy results. Our results may help optimize protocols for better investigation of molecular mechanisms in physiological and pathological conditions in male turkey breeders for further improving commercial flock welfare and livestock production, and are a prerequisite for future studies determining YSS etiology.

## Introduction

Yellow semen syndrome (**YSS**) is an endemic condition in male breeder turkeys characterized by the production of semen with a yellow color and an increased total protein concentration in seminal plasma, giving poor fertility and hatchability outcomes (Thurston et al., 1992). Research on YSS etiology, especially with molecular biology techniques, has been scarce, but is of considerable value, contributing to the appropriate management of YSS individuals in commercial breeding farms. A useful tool for diagnostic purposes for its high sensitivity, repeatability, and reliability, with a relatively simple methodology, is quantitative PCR (**qPCR**), previously successfully used in poultry science for serotyping and identifying virulence factors in commercial flocks. Molecular biology methods, including qPCR, may be helpful in research determining the etiology of YSS. Nevertheless, to ensure high-reliability results in qPCR studies, internal controls and normalization against at least two carefully selected reference genes are required (Thellin et al., 2009).

In the current study, we aimed to indicate the best pair of reference genes for qPCR experiments in male turkeys’ reproductive tract (testis, epididymis, ductus deferens), liver, and immune tissues, and cells i.e. bursa of Fabricius, thymus, whole blood, peripheral blood mononuclear cells (**PBMC**), and spleen of healthy adult individuals producing white semen (**WS**), and across WS and YSS groups to provide adequate internal controls for YSS studies. For this, we selected seven commonly used housekeeping genes, i.e., *glyceraldehyde-3-phosphate dehydrogenase* (***GAPDH***), *actin beta* (***ACTB***), *phosphoglycerate kinase* (***PGK1***), *60S ribosomal protein L13* (***RPL13***), *ribosomal protein L19* (***RPL19***), *transferrin receptor protein* (***TFRC***), and *vimentin* (***VIM***), and performed qPCR followed by computational calculation of these genes' expression stability in selected tissues/cells. Our results are a prerequisite for future studies determining YSS etiology and related studies.

## Material and methods

### Ethical statement

Following the Act of 15 January 2015 on the Protection of Animals Used for Scientific or Educational Purposes and Directive 2010/63/EU of the European Parliament and the Council of 22 September 2010, Article No 2.1.6 on the Protection of Animals Used for Scientific or Educational Purposes, the ethical evaluation and approval were waived for this study, since all of the experiments were conducted on animal tissues collected post-mortem.

### Animals, group characteristics, and sample collection

Semen was collected by abdominal massage from turkey toms BUT6 (32-36 weeks old, Aviagen Turkeys, Tattenhall, Cheshire, Great Britain) from Zdrowy Drob Ltd. in Frednowy, Indykpol, Drosed Group (Poland). Semen samples were examined for sperm motility using computer-assisted sperm analysis, and sperm concentration and viability on Muse Cell Analyzer (EMD Millipore, Billerica, USA), according to the manufacturer. Seminal plasma was obtained by double centrifugation of fresh semen (10 min at 4 °C; 9,000 × *g*), and total protein in seminal plasma was measured using the BCA protein assay kit (#23225, ThermoFisher Scientific, Waltham, MA, USA) following a manufacturer's protocol. Based on Thurston et al.’s (1992) criteria, individuals were classified into white semen (WS)-producing toms (WS; *n* = 6; total protein in seminal plasma < 20 mg/mL) or yellow semen (YS)-producing toms (YSS; *n* = 6; total protein in seminal plasma > 55 mg/mL).

Three days after semen collections and classifications of WS- and YS- YS-producing turkeys, birds were slaughtered. The peripheral blood was collected during slaughter into sterile Eppendorf tubes, mixed 1:1 with Tri Reagent® (#T9424, Sigma Aldrich, Madison, USA), snap-frozen in liquid nitrogen, and considered whole blood samples. For isolation of PBMC, the peripheral blood was collected into sterile 50 ml tubes containing 1000 μL of 0.3 M EDTA (#879810112, POCH, Gliwice, Poland) and 1 % acetylsalicylic acid, pH 7.4 (#A2093, Sigma-Aldrich, Madison, USA). Fragments of testis, epididymis, ductus deferens, liver, bursa of Fabricius, thymus, and spleen were collected, washed with sterile ice-cold phosphate-buffered saline, pH = 7.4 (**PBS**, #P4417, Merck, USA), and snap-frozen in liquid nitrogen.

### PBMC isolation

Blood samples stabilized with ethylenediaminetetraacetic acid and acetylsalicylic acid were proceeded immediately after slaughter. First, samples were diluted (1:1) with Hank's balanced salts, pH = 7.4 (#H1387, Merck, USA) containing 1 % of antibiotic-antimycotic solution (**AB**, #5955, Merck, USA), and overlaid on Ficoll-Paque PLUS (density 1.077 g/mL; #17144002, Cytiva, USA) in proportion 3:2. Centrifugation was held 20 min, 10°C, 600 × *g*, minimal acceleration and deceleration values. A formed buffy coat containing PBMC fraction was aspired, and washed twice in PBS containing 1 % AB for 12 min, at 4°C, 600 x g. Cell viability was tested with trypan blue staining method (#T8154, Merck, USA), and was above 95 %. Cells were counted and frozen at – 80°C in a concentration of 60 million cells/mL of fetal bovine serum (#10270106, ThermoFisher Scientific, USA) with 10 % dimethyl sulfoxide (#D2650, Merck, USA).

### RNA extraction

RNA extraction was performed according to the protocol relevant to TRI Reagent®. All samples were kept frozen until immersion in TRI Reagent®. The whole blood samples containing TRI Reagent® (1:1) added before freezing in liquid nitrogen (−196°C), were added to a fresh portion (200 µL) of TRI Reagent®. PBMC samples (60 million cells) were washed with sterile PBS with 1 % AB and poured with 200 µL TRI Reagent®. A 20 ± 5 mg weighing fragments of testis, epididymis, ductus deferens, liver, bursa of Fabricius, thymus, and spleen were first immersed in 500 µL of TRI Reagent® and homogenized on ice using a TissueRuptor II (Qiagen, USA). In the case of liver and whole blood samples, RNA binding and purifying was done on RNeasy Mini spin columns according to the RNeasy® Mini Kit (#74104, Qiagen, USA) protocol. RNA aliquots were used for RNA quality control (optical density, A260/A280), and determination of RNA concentration using a DS-11 spectrophotometer (DeNovix, USA), and stored at −80°C until further used for cDNA synthesis and qPCR.

### cDNA synthesis and qPCR

RNA aliquots were first treated with a DNA wipe-out buffer containing Dnase I (DNase 1 Amplification Grade, AMPD1-1KT, #051M6157, Sigma Aldrich, Madison, USA), following a manufacturer's protocol, to eliminate potential contamination with genomic DNA. Afterward, cDNA was synthesized using a High-Capacity cDNA Reverse Transcription Kit (#4368814, Applied Biosystems, USA), following a manufacturer's protocol, and stored at −20°C until further use.

The amplification was conducted in a Viia7 system (Applied Biosystems). The reaction was held in duplicates, and the mixture for the qPCR assay consisted of 3 μL DNA (10 ng cDNA for testis, epididymis, ductus deferens, bursa of Fabricius, spleen, and liver; and 50 ng cDNA for thymus, whole blood, and PBMC, selected during the optimization step, data not shown), 5 μL Premix *Ex Taq*™ (2x) (Probe qPCR) Bulk, 0.02 μL ROX Reference Dye (both #RR390L, Takara Bio INC, Japan), 0.5 μL custom-designed TaqMan probes (Thermo Fisher Scientific) listed in Table 1, and 1.48 μL nuclease-free water (#129114, Qiagen, USA) to a final volume of 10 μL. Amplification was held as follows: initial denaturation (30 s at 95°C), 60 reaction cycles of denaturation for 5 s at 95°C, and annealing for 30 s at 60°C. A no template control and no reverse transcriptase control were performed on each set of reactions.

**Table 1:** TaqMan probes used for reference gene selection for gene expression analyses in reproductive turkey (*Meleagris gallopavo*) with yellow semen syndrome.

**Gene symbol**	Gene name	Accession number	Assay ID	Mean efficiency
***RPL13***	*60S ribosomal protein L13*	XM_010718177.1	AP33DMH	84 %
***TFRC***	*Transferrin receptor protein*	XM_003209136.4	AP4767F	82 %
***RPL19***	*Ribosomal protein L19*	XM_010724597.3	AP7DZTD	79 %
***VIM***	*Vimentin*	XM_010712706.3	AP9HVDA	83 %
***GAPDH***	*Glyceraldehyde-3-phosphate dehydrogenase*	NM_001303179.1	APAAKPZ	88 %
***ACTB***	*Actin beta*	NM_001303173.1	APCFFAX	78 %
***PGK1***	*Phosphoglycerate kinase*	XM_010715363.3	APDJ9VV	83 %

The cycle threshold (**Ct**) values were used to calculate the best combination of reference gene pairs for each studied material of healthy adult individuals producing WS, and across WS and YSS groups to provide adequate internal controls for YSS studies, using the RefFinder online tool (Xie et al., 2012) integrating current major computational programs i.e., comparative ΔCt method (Silver et al., 2006), BestKeeper (Pfaffl et al., 2004), NormFinder (Andersen et al., 2004), and geNorm (Vandesompele et al., 2002).

## Results and discussion

**Fig. 1:**
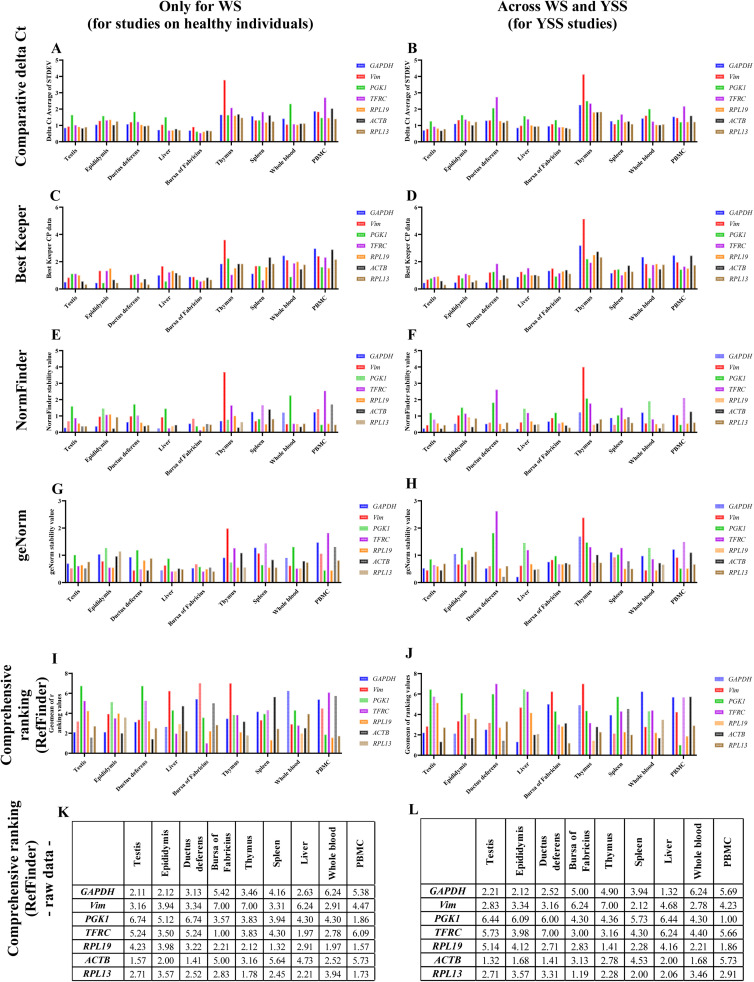
A graphical presentation of the summary results of comparative delta Ct analysis (A, B), BestKeeper (C, D), NormFinder (E, F), geNorm (G, H), and over final ranking (RefFinder) (I-K) of selected reference genes expression stability in white semen producing turkey toms (A, C, E, G, I, K) and respecting yellow semen syndrome occurrence (B, D, F, H, J, L). A lower value calculated for each gene is associated with higher expression stability.

The present study evaluated the expression stability of seven commonly used housekeeping genes – *ACTB, GAPDH, RPL13, RPL19, VIM, PGK1*, and *TFRC –* in the reproductive tract, liver, and immune tissues, as well as in cells isolated from adult male breeder turkeys producing white semen (i.e., high-quality semen), and across WS and YSS individuals, which is essential to conduct studies on YSS pathogenesis. Summary results are presented in Fig. 1. Generally, a lower value calculated for each gene is associated with higher expression stability. We have provided recommendations for 1/ WS turkey tomes, which might be interesting for future studies on physiological processes occurring in healthy individuals (Fig. 1A, C, E, G, I, K), and 2/ for WS and YSS turkey tomes – across groups – for further studies on YSS pathogenesis (Fig. 1B, D, F, H, J, L). Notably, the only parameter distinguishing WS and YSS individuals used in the current study was total protein concentration in seminal plasma, i.e. 16.25 ± 3.07 mg/mL vs 99.17 ± 27.21 mg/mL in WS and YSS individuals, respectively (*P* < 0.0001). The semen volume (0.26 ± 0.08 mL vs 0.22 ± 0.10 mL), sperm concentration (9.13 ± 2.13 × 10^9^/mL vs 6.98 ± 1.33 × 10^9^/mL), viability (93.02 ± 1.96 % vs 93.97 ± 1.67 %), and motility (71.73 ± 1.95 % vs 70.68 ± 10.6 %) did not differ (*P* > 0.05) between WS and YSS turkey tomes, respectively. Also, the age range was limited to ensure the exclusion of age or season effect. For this reason, we believe that our recommendations might be used as first-choice reference genes in further studies, especially on YSS. Nevertheless, additional studies should assess a wider age range for reference genes to confirm their usefulness for the entire semen production cycle and tom age.

Our current results show that for the reproductive tissues, the most stable reference gene pair was *ACTB* and *GAPDH*. The only exception was the ductus deferens of WS-producing toms, where *RPL13* appeared more stable than *GAPDH.* Notably, the wide functional diversity, including being a key glycolytic enzyme, makes GAPDH essential for proper cellular metabolic activity maintenance, guaranteeing its ubiquitous expression. Similarly, *ACTB* encoding for *β*-actin, is a highly conserved protein in vertebrates, an essential component of the cytoskeleton, and an element of key cellular processes. Despite that many studies confirm that both, *ACTB* and *GAPDH* may possess a marked expression variability between different tissue types (Lin and Redies, 2012), according to Słowińska et al. (2020), the expression of *ACTB* and *GAPDH* does not vary between tissues of the adult male turkey reproductive tract (Słowińska et al., 2020), which is in line with our current results (Fig. 1).

In the case of the liver, in WS turkey toms the most stable was the *ACTB/TFRC* gene pair, whereas when the analysis was performed across experimental groups (WS and YSS together for YSS studies), a more relevant internal control would be the *ACTB/GAPDH* gene pair (Fig. 1). Notably, *TFRC* and *RPL13* were previously recommended as priority candidates for gene expression studies in turkey liver in different infection models (Mitra et al., 2016). Thus, *TFRC* and *RPL13* might be interesting first-choice candidates for qPCR normalization in turkeys’ liver in several experimental conditions. However, in the case of studies respecting potential YSS-related alterations in the liver, we recommend using the *GAPDH* as the first-choice candidate for qPCR normalization.

In the thymus, the highest expression stability was assigned to the *RPL13/RPL19* gene pair, both for WS-producing tomes and concerning YSS occurrence. In the bursa of Fabricius, the highest expression stability was calculated for *RPL13, RP19*, and *TFRC*, and only slight differences occurred regarding YSS occurrence. The most stable reference gene pairs in the spleen, whole blood, and PBMC were *RPL19/RPL13, RPL19/ACTB*, and *RPL19/RPL13*, respectively, for WS-producing toms, and *RPL13/VIM, ACTB*/*RPL19*, and *PGK1/RPL19*, respectively, considering YSS occurrence. The RPL19 is a cytoplasmic component of the large ribosomal subunit, and thus, involved in protein synthesis in the cell. For this reason, its ubiquitous expression of *RPL19* is undeniable. Our current study showed that *RPL19* topped the stability ranking in whole blood and PBMC samples of WS-producing toms, was in second place in this ranking for respecting YSS occurrence, and was highly stable in the studied central and peripheral immune organs. This highlights the accuracy of selecting *RPL19* as the first choice reference gene in studies involving gene expression analysis in the immune system of adult breeder turkeys, even respecting YSS occurrence.

The reference genes recommended in the present study should undergo further validation to confirm their stability within each planned experiment. Nevertheless, they may be recognized as first-choice candidate reference genes for molecular biology experiments investigating reproductive, metabolic, and immune mechanisms in male breeder turkeys, especially respecting YSS occurrence in commercial flocks. Our recommendations may help optimize protocols for better investigation of molecular mechanisms in physiological and pathological conditions in male turkey breeders for further improving commercial flock welfare and livestock production, also concerning YSS occurrence.

## Disclosures

The authors declare that they have no known competing financial interests or personal relationships that could have appeared to influence the work reported in this paper.

During the preparation of this work, the author(s) used Grammarly and ChatGPT to correct the language. After using these tools, the author(s) reviewed and edited the content as needed and take(s) full responsibility for the content of the publication.
